# Genome-Wide Investigation of *CPK-Related Kinase* (*CRK*) Gene Family in *Arabidopsis thaliana*

**DOI:** 10.3390/ijms26073297

**Published:** 2025-04-02

**Authors:** Shiquan Yang, Yuan Fang, Xianming Fang, Jingwen He, Kai He

**Affiliations:** Ministry of Education Key Laboratory of Cell Activities and Stress Adaptations, School of Life Sciences, Lanzhou University, Lanzhou 730000, China; yangshq20@lzu.edu.cn (S.Y.); fangy2024@lzu.edu.cn (Y.F.); xmfang@lzu.edu.cn (X.F.); hejw2019@lzu.edu.cn (J.H.)

**Keywords:** kinase, calcium, phosphorylation, CRK, CPK, signal transduction

## Abstract

Calcium-dependent protein kinase (CPK), representing a group of typical Ca^2+^ sensors in plants, has been well characterized in plants. CPK is capable of binding to Ca^2+^, which sequentially activates CPK. CPK-related kinase (CRK) shows protein structures similar to CPK but only contains degenerative EF-hands, which likely makes the activation of CRK Ca^2+^ independent. Compared with CPK, CRK is barely functionally analyzed. In this study, we systematically investigated *CRK* genes in the Arabidopsis genome. We found that *CRK* appeared to emerge in land plants, suggesting *CPK* and *CRK* are divided at very early stages during plant evolution. In Arabidopsis, the detailed analysis of the calmodulin-like domain of CRK indicated the substitutions of key amino acid residues in its EF-hands result in disrupted Ca^2+^ association. Next, by using a YFP tag, we found that all Arabidopsis CRK proteins were localized at the plasma membrane. After cloning the promoters of all eight *CRK* genes, we found that *CRK*s were widely expressed at all stages of Arabidopsis by using GUS staining. Furthermore, the kinase activity of CRK was examined by using phospho-antibody and Pro-Q staining. CRK was shown to possess high autophosphorylation, which was not affected by the presence of Ca^2+^. Moreover, we analyzed the cis-elements of *CRK* promoters and discovered that stress signals potentially regulate the expression of *CRK* genes. Consistently, by using quantitative real-time PCR (qPCR), we found a number of *CRK* genes were regulated by a variety of biotic and abiotic treatments such as flg22, ABA, drought, salt, and high and low temperatures. Furthermore, by utilizing proteomic approaches, we identified more than 100 proteins that interacted with CRK5 *in planta*. Notably, RLK and channels/transporters were found in CRK5-containing complexes, suggesting they function upstream and downstream of CRK, respectively.

## 1. Introduction

Plants employ multiple strategies to transduce external and internal cues into cellular signaling to coordinate growth/development and stress responses, in which protein modifications play a central role [1]. The first characterized protein modification was phosphorylation, which is mediated by protein kinases. Kinases transfer the γ-phosphate group of ATP to the threonine (Thr/T), serine (Ser/S), and tyrosine (Tyr/Y) residues of substrate proteins, resulting in the reforming of charge effects and subsequent conformational changes [2]. The phosphorylation made to proteins causes altered protein features such as enzymatic activities, stabilities, subcellular locations, and binding affinities, leading to reprogramming downstream signaling events and, ultimately, cellular responses [3].

In plants, Ca^2+^ not only serves as an essential micronutrient but also acts as a crucial signal molecule. Ca^2+^ signals are encoded by plasma membrane (PM) or endomembrane-localized Ca^2+^ channels and transporters. Specific stimuli cause unique Ca^2+^ oscillations, referred to as Ca^2+^ signatures. The Ca^2+^ signatures are next decoded by a group of Ca^2+^-binding proteins known as Ca^2+^ sensors, such as calmodulin (CaM), calcineurin B-like protein (CBL), and calcium-dependent protein kinases (CPK) [4]. CaM contains four EF-hands that are directly associated with Ca^2+^. Both CBL and CPK have CaM-like domains that possess Ca^2+^-binding affinity [5].

In plants, CPK belongs to a kinase super-family also containing sucrose non-fermenting 1 (SNF1)-related kinase 1 (SnRK1), SnRK2, SnRK3, and CPK-related kinase (CRK) [6]. The SnRK1 homologs are known as SNF1 and AMP-activated protein kinase (AMPK) in yeast and animals, respectively. SnRK1/SNF1/AMPK play central roles in sugar sensing and energy homeostasis [7,8]. In plants, *SnRK1* is required for normal growth as the *snrk1*.*1 snrk1*.*2* double mutant is lethal in Arabidopsis [9]. Moreover, *SnRK1* engages in various stress responses [10]. In plants, *SnRK1*/*SNF1*/*AMPK* genes are expanded and form a super-family during evolution. *SnRK2*, *SnRK3*, *CPK*, and *CRK* are only found in plant genomes. *SnRK2* was originally found to mediate abscisic acid (ABA) signaling [11]. SnRK2 is inhibited by protein phosphatase 2C (PP2C), while it is activated by the presence of ABA [12]. The activation of SnRK3, also known as CBL-interacting protein kinase (CIPK), depends on Ca^2+^. In the presence of Ca^2+^, CBL interacts with and activates CIPK. Unlike CIPK, which does not directly bind to Ca^2+^, CPK interacts with Ca^2+^ to release the inhibition caused by its autoinhibitory domain. This interaction allows CPK to activate its kinase domain. Thus, although both CIPK and CPK are involved in calcium signaling, they function by different mechanisms: CIPK relies on CBL for activation, whereas CPK binds directly to Ca^2+^ to reach its active form [13,14]. CPK phosphorylates downstream components to transduce cellular signaling.

CRK shares high similarities with CPK in terms of protein structures. However, degenerative EF-hands are found in the CaM-like domain of CRK, which makes it unlikely that CRK is activated by Ca^2+^ [15]. CRK was first cloned and named in maize and found in rice thereafter [16,17]. Biochemical assays demonstrated that the kinase activity of CRK in maize was not regulated by Ca^2+^ [18]. In tobacco, the overexpression of *NtCBK1* (*CRK*) results in a late flowering phenotype [19]. In tomatoes, *SlCRK1* plays a role in the ethylene and salicylic acid (SA) signaling pathways for mechanical and cold responses [20]. The *slcrk6* mutant exhibits increased susceptibility to *PstDC3000* [21]. A recent study revealed that ZmCRK1 phosphorylates H^+^-ATPase to regulate drought response in maize [22]. In Arabidopsis, *CRK1* has been implicated in regulating salt adaption [23]. The *crk1* mutant exhibits a semi-dwarf phenotype under continuous light conditions [24]. CRKs phosphorylate tyrosine residues to modulate SA, gibberellic acid (GA), and ABA signaling pathways [25,26,27]. CRK3 phosphorylates the cytoplasmic glutamine synthetase GLN1;1 to regulate leaf senescence [28]. Additionally, CRK3 phosphorylates HSFA1a, a heat shock protein, to be involved in heat responses [29]. CRK5 was shown to be localized to PM to phosphorylate PIN2, accelerating exocytosis through BFA-sensitive membrane recycling [30,31,32]. Moreover, the degradation of CRK5 is regulated by WDRP, a DDB1-binding WD40 protein, in a ubiquitin-dependent manner [33]. In summary, the studies of *CRK* genes indicate that *CRKs* are involved in plant growth/development and stress responses. However, compared to *CPK*, the detailed molecular mechanisms underlying *CRK* functions are far less studied. For instance, the upstream and downstream components of CRKs remain largely elusive and await further investigations.

In this study, we systematically investigated all eight *CRK* genes in Arabidopsis. By analyzing *pCRK*::*GUS* plants, we found *CRK* genes were widely expressed in all stages of a life span. CRK proteins appeared to be localized to PM, even though not all CRKs contain N-terminal lipidation sites. The D-x-D motif in EF-hands that is essential for Ca^2+^-binding is missing in CRK, which likely causes CRK to lose its Ca^2+^-association ability. The biochemical results confirmed that CRK activation is Ca^2+^-independent. *CRK* genes were found to respond to different stressed conditions using quantitative real-time PCR (qPCR). Over 100 candidate proteins were identified as CRK5-interacting components, including receptor-like kinase (RLK) and channels/transporters, such as SERK4 and STP1. This study aimed to provide a survey of *CRK* genes in Arabidopsis, which potentially contributes to the future functionality of *CRK* genes.

## 2. Results

### 2.1. CRK Represents a Group of CPK-like Kinase That Contains Degenerative EF-Hands

**Figure 1:**
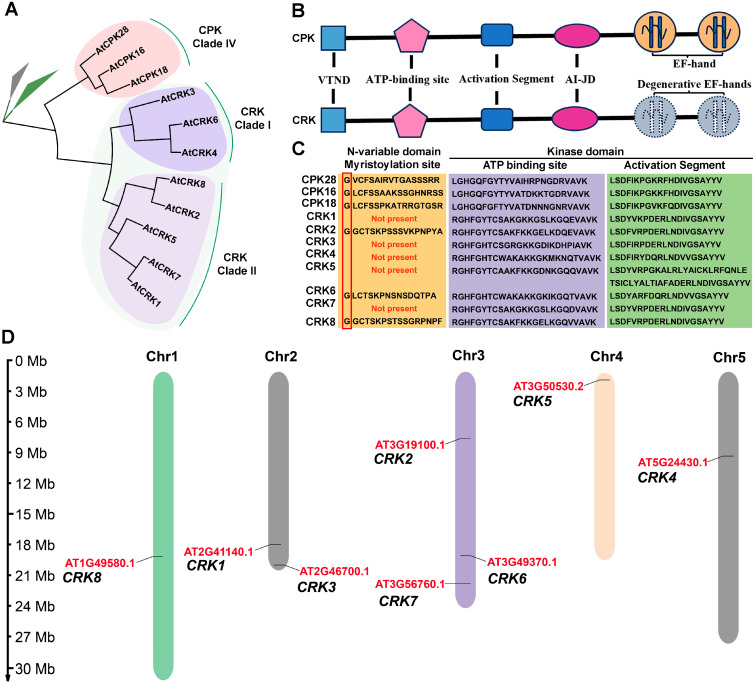
Phylogenetic and domain structural analyses of CRK. (**A**) Maximum likelihood phylogeny of Arabidopsis CRK based on the protein sequences by using MEGA11. (**B**) The domain structures of CRK and CPK. CRK and CPK consist of a variable N-terminal domain (VNTD), an activation segment, an auto-inhibitory junction domain (AI-JD), and a C-terminal CaM-like domain with functional (CPK) or degenerative (CRK) EF-hands. (**C**) Multiple sequence alignment of CRK and CPK protein sequences. The myristoylation sites of CRK and CPK are highlighted in red. (**D**) Chromosomal distributions of *CRK* genes in the genome of *Arabidopsis thaliana*.

Eight *CRK* genes, namely *CRK1* to *CRK8*, are identified in the Arabidopsis genome. The *CRK* gene family is comprised of two subclades. Clade I consists of *CRK3*, *4*, and *6*, and Clade II contains *CRK1*, *2*, *5*, *7*, and *8* (Figure 1A). CRK shows protein structures similar to CPK. The *CPK* subclade IV, consisting of *CPK16*, *18*, and *28*, is the closest paralog of *CRK* (Figure 1A). Both CRK and CPK contain a variable N-terminal domain (VTND), a kinase domain, an auto-inhibition junction domain (AI-JD), and a CaM-like domain (Figure 1B). The VTND of a number of CPKs and CRKs contains the G-x-x-x-S/T motif, which is the recognition site for N-myristoylation (Figure 1C). The N-terminal myristoylation often determines the membrane association of the modified proteins. The kinase domains of both CRK and CPK possess an ATP-binding site, associating with ATP, and an activation segment, which regulates the kinase activity (Figure S1). AI-JD functions to inhibit the kinase domain when Ca^2+^ is absent. When Ca^2+^ binds to EF-hands, the inhibition of AI-JD on the kinase domain is released by the interaction of AI-JD and EF-hands, resulting in CPK activation. Distinct from CPK, CRK merely contains degenerative EF-hands in its CaM-like domain, suggesting the kinase activation of CRK is Ca^2+^-independent.

### 2.2. Distribution of CRK Genes and Analysis of the Physicochemical Properties of CRK Proteins

We analyzed the chromosomal distribution of *CRK* genes. *CRK8* is located on chromosome one, *CRK1*/*3* are located on chromosome two, *CRK2*/*6*/*7* are located on chromosome three, *CRK5* is located on chromosome four, and *CRK4* is located on chromosome five (Figure 1D). In addition, the physicochemical properties of CRK proteins were predicted. CRKs contain amino acid residues ranging from 500 to 600, with molecular weights ranging from 60 to 70 kDa (Table 1). The theoretical pIs, instability indexes, and aliphatic indexes of CRKs are also predicted (Table 1).

### 2.3. CRK Widely Exists in Land Plants

**Figure 2:**
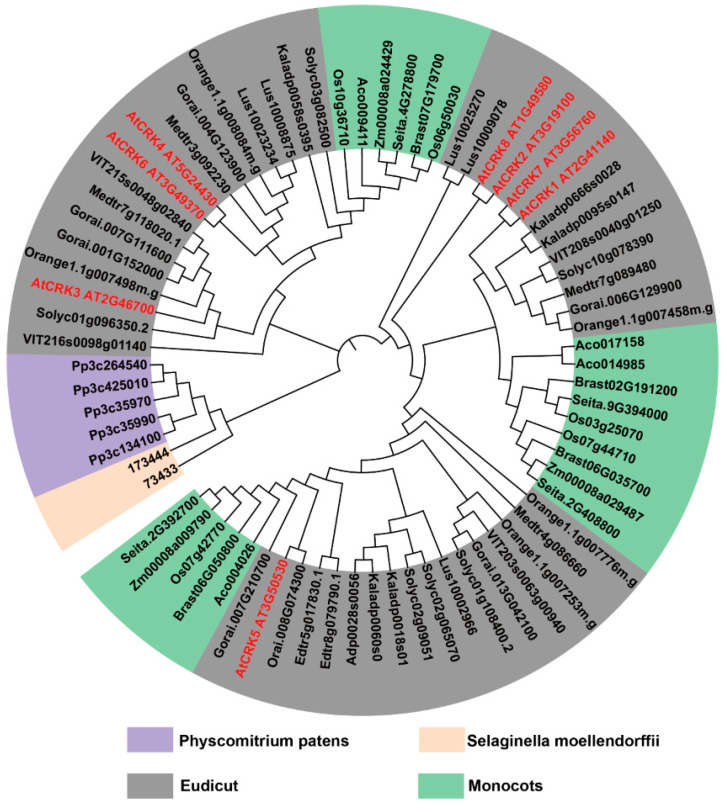
The evolutionary tree generated based on plant CRK. Phylogenetic analysis of Arabidopsis CRK (red) and CRK in other plants through the maximum likelihood method with 1000 bootstraps by using MEGA11. The phylogenetic groups of CRK were marked in different colors.

To resolve the evolutionary relationships among the *CRK* genes in diverse plants, we performed a maximum-likelihood analysis of the conserved CaM-like domains in CPK and CRK. *CRK* genes are found in land plants but not in algae *Chromochloris zofingiensis and Chondrus crispus*, while *CPK* is identified in plants, including algae (Figure 2 and Figure S2A,B). It is suggested that Ca^2+^-mediated regulations emerge at the early stages of plant evolution. In mosses and vascular plants, CRK clusters form a clade. In angiosperms, CRK does not show a trend of lower to higher evolution (Figure 2). These results suggest that the divergence of *CRK* and *CPK* likely occurred when ancient plants relocated from the oceans to the lands.

### 2.4. CRK Loses the Ability to Associate with Ca^2+^

**Figure 3:**
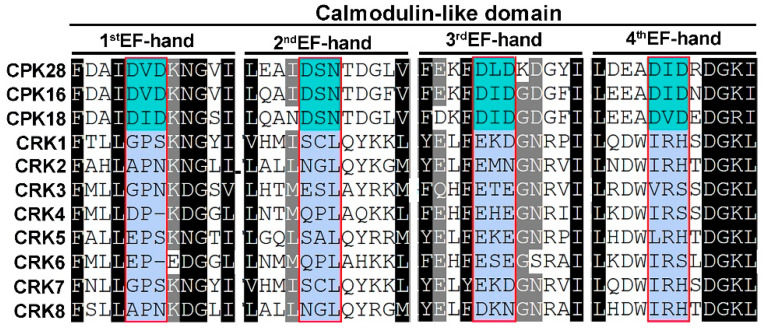
The EF-hands of CRK and CPK in Arabidopsis. Multiple sequence alignment of C-terminal sequences of CRK and CPK proteins in Arabidopsis. CPK contains EF-hand domains with conserved D-x-D motifs (x is any amino acid), which are crucial for calcium binding. CRK has no conserved D-x-D motifs in its predicted EF-hands. Medium turquoise indicates conserved D-x-D motifs in CPK, light steel blue indicates degenerative EF-hands in CRK.

**Figure 4:**
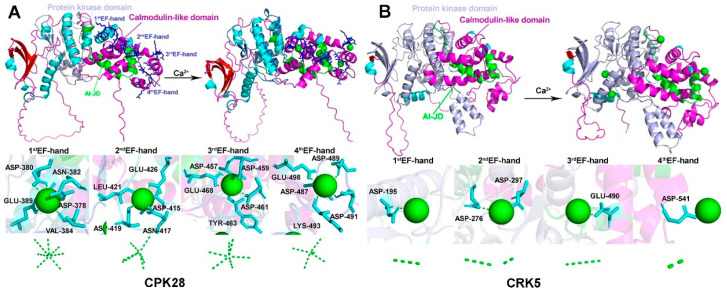
Molecular structures of CaM-like domains in CRK and CPK. (**A**) Potential H-bonds that bind to Ca^2+^ in the EF-hands of CPK28. Three-dimensional structure of the CPK28 protein with Ca^2+^ ligand binding to the EF-hands. Green broken lines denote H-bonds. (**B**) Potential H-bonds that bind to Ca^2+^ in the EF-hands of CRK5. Three-dimensional structure of the CRK5 protein with Ca^2+^ ligand binding to the EF-hands. Green broken lines denote H-bonds. The molecular models were constructed using AlphaFold3 (https://golgi.sandbox.google.com/) URL (accessed on 23 March 2025).

We analyzed the CaM-like domain of CRK in Arabidopsis and compared it to CPK, which contains four EF-hands, in which the D-x-D motif contributes to direct Ca^2+^-binding (Figure S3). The amino acid substitutions in the D-x-D motif of CRK potentially disrupt Ca^2+^ association (Figure 3 and Table 2). The notion was further tested by using AlfaFold3. In CPK28, five to seven hydrogen (H)-bonds contribute to Ca^2+^-binding. For instance, the amino acid residues in the first EF-hand (D378, D380, N382, V384, E389), second EH-hand (D415, N417, D419, L421, E426), third EH-hand (D457, D459, D461, Y463, E468), and fourth EH-hand (D487, D489, D491, K493, E498) directly associated with Ca^2+^ via non-convent H-bonds (Figure 4A). By contrast, one to two hydrogen (H)-bonds were found to potentially associate with Ca^2+^ in each EF-hand of CRK5 (D195, D276, D279, E490, D541) (Figure 4B), indicating CRK is not likely to directly bind to Ca^2+^.

### 2.5. The Subcellular Localizations of CRK

**Figure 5:**
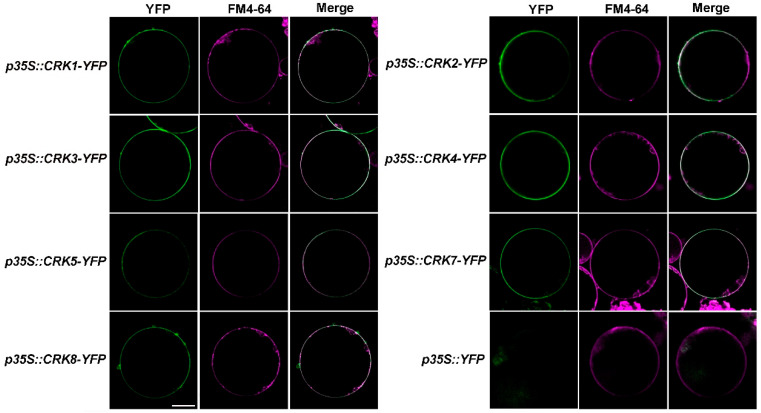
Subcellular localizations of CRK proteins. The subcellular localizations of CRK proteins were detected by transiently expressed *CRKs-YFP* in *N. benthamiana* protoplasts. Scale bars, 25 μm. *p35S::YFP* was used as the control.

Next, the subcellular locations of Arabidopsis CRK were analyzed. Except for *CRK6*, which was unable to be cloned yet, likely due to extremely low expression, all seven *CRK* genes were cloned and fused with a *YFP* tag. The CRK-YFP plasmids were transiently expressed in *Nicotiana Benthamiana* cells, followed by protoplast isolation. Even though only CRK2, 6, and 8 appeared to possess N-myristoylation site (Figure 1C), all *CRKs*-*YFP* were found to be PM-associated (Figure 5). This indicated that CRK, similar to CPK, likely functions as a modulator in regulating membrane proteins such as channels and transporters.

### 2.6. The Expression Patterns of CRK Genes in Arabidopsis

**Figure 6:**
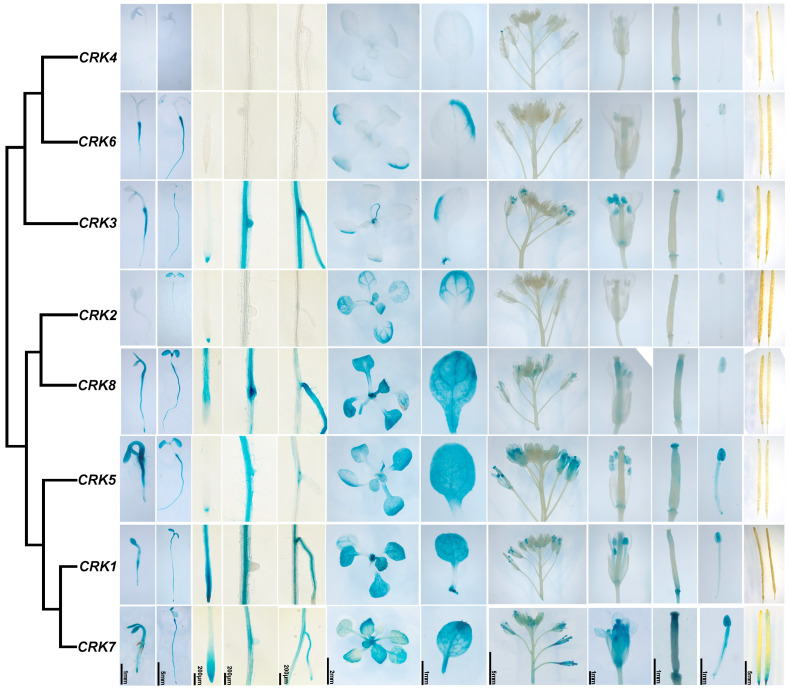
Tissue-specific expression of CRK genes. *Pro*::*GUS* transgenic plants were generated for all eight Arabidopsis *CRK* genes. Seedlings and tissues were stained with X-Gluc for GUS expression analysis. From left to right, a 2-day-old seedling after germination, a 4-day-old seedling, a root tip from an 8-day-old seedling, an early lateral root from an 8-day-old seedling, a late lateral root from an 8-day-old seedling, a shoot from a 14-day-old seedling, a leaf from a 14-day-old seedling, an inflorescence from a 35-day-old plant, a mature flower from a 35-day-old plant, and a mature silique from a 35-day-old plant.

To analyze the expression patterns of *CRK* genes at the tissue levels, we cloned the promoter regions of all eight *CRK* genes. The transgenic plants harboring *pCRK*::*GUS* were generated. The expression patterns of *CRK* were examined by using GUS staining. Except for *CRK4*, which showed very low expression during all developmental stages, all other *CRKs* were found to be globally expressed (Figure 6). *CRK2*, *3*, and *5* were expressed in the root tip, and *CRK1*, *3*, *7*, and *8* were expressed in the lateral root (Figure 6). *CRK1*, *2*, *5*, *7* and *8* showed strong expression in the leaf. *CRK1*, *3*, *5*, and *7* were obviously expressed in floral organs, including pollen tubes (Figure 6).

**Figure 7:**
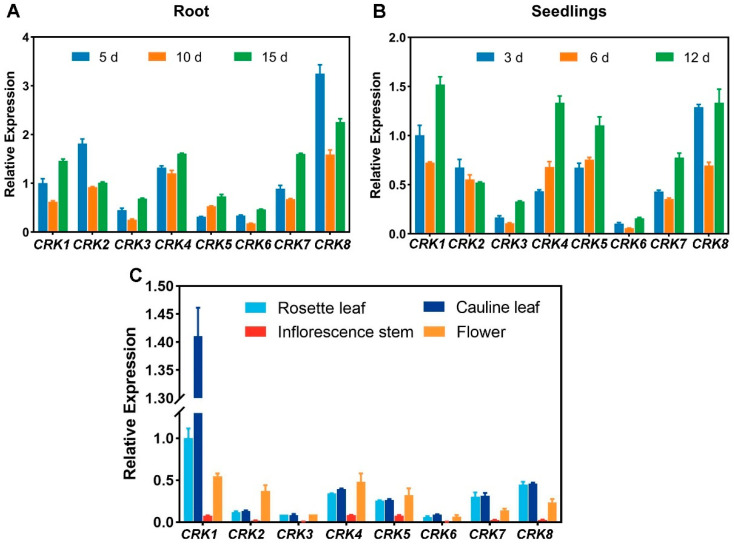
RT-qPCR analysis of the expression of *CRK* genes in various developmental stages and different tissues. (**A**) Relative expression of *CRK* genes in the root of 5-, 10-, and 15-day-old plants was quantified by RT-qPCR. (**B**) Relative expression of *CRK* genes in 3-, 6-, and 12-day-old seedlings was quantified by RT-qPCR. (**C**) Relative expression of *CRK* genes in 21-day-old rosette leaf, cauline leaf, 35-day-old inflorescence stem, and flower was quantified by RT-qPCR.

We also utilized RT-qPCR to verify the expression patterns of *CRK*s. Consistent with GUS staining results, *CRK1* and *8* showed high expression in roots (Figure 7A). In seedlings, *CRK1*, *5*, and *8* were highly expressed (Figure 7B). *CRK1* displayed much higher expression levels than other *CRKs* in the leaf (Figure 7C). The expression of CRK*1*/*7*/*8* showed a V-shaped pattern, suggesting these genes were finely regulated during these times of development and were potentially important for these stages of growth and development. High-order mutants of these *CRK* genes can be generated and analyzed in future studies.

### 2.7. The Kinase Activity of Arabidopsis CRK Is Ca^2+^-Independent

**Figure 8:**
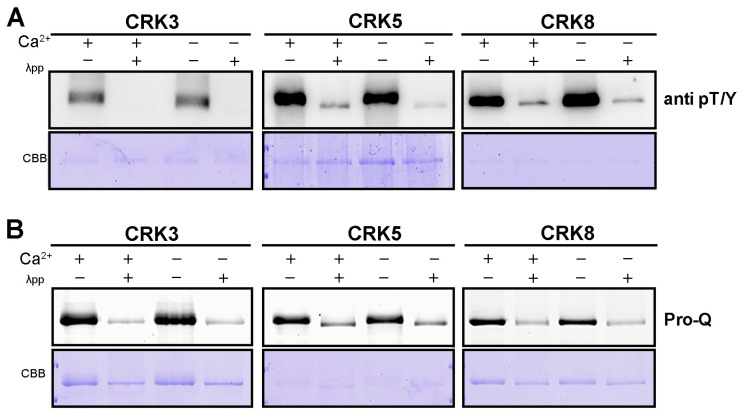
Arabidopsis CRK has Ca^2+^-independent kinase activity. (**A**) The autophosphorylation levels of CRKs. CRKs fused with MBP-tag were purified and examined. Phosphorylated proteins were separated by SDS-PAGE gels and detected by using a phospho-T/Y antibody (upper panels). Total proteins were Coomassie Brilliant Blue (CBB) stained in SDS-PAGE gels (bottom panels). (**B**) In vitro kinase assay using Pro-Q staining showed the autophosphorylation of CRKs.

It is well established that the kinase domain of Arabidopsis CPK is activated upon Ca^2+^ association. However, it was unknown whether Ca^2+^ is required for CRK activation in Arabidopsis. By using an α-phospho T/Y antibody, we were able to detect the autophosphorylation of *CRK3*, *5*, and *8*, which was eliminated by the addition of phosphatase λpp (Figure 8A). Of note, this phosphorylation of CRK was spotted without the presence of Ca^2+^, and adding Ca^2+^ failed to promote CRK phosphorylation (Figure 8A). Furthermore, the phosphorylation levels of CRK were examined by using Pro-Q. Consistent with Western immunoblotting results, the Pro-Q staining showed that the phosphorylation of *CRK3*, *5*, and *8* was Ca^2+^-independent (Figure 8B). These results thus demonstrated that the kinase activity of Arabidopsis CRK is spontaneous, in which Ca^2+^ is not required.

### 2.8. The Analysis of Cis-Elements of the Promoters of CRK

**Figure 9:**
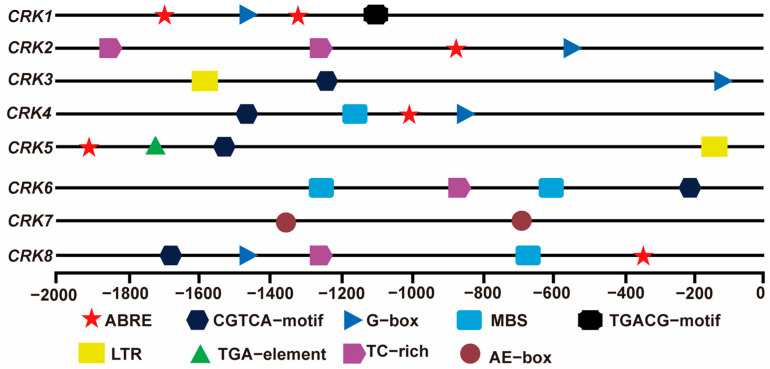
Analysis of cis-acting regulatory elements in the promoter regions of *CRK* genes. ABRE: cis-acting element involved in the abscisic acid responsiveness. CGTCA-motif and TGACG-motif: cis-acting regulatory element involved in the MeJA-responsiveness. G-box: cis-acting regulatory element involved in light responsiveness. TGA-box: part of an auxin-responsive element. LTR: cis-acting element involved in low-temperature responsiveness. TGA-element: auxin-responsive element. MBS: MYB binding site involved in drought-inducibility. TC-rich: cis-acting element involved in defense and stress responsiveness. AE-box: part of a module for light response.

We next analyzed the promoter regions of *CRK* genes. Among all the cis-elements, most *CRK* promoters contained conserved ABRE-motif and G-box cis-elements. A number of *CRK* promoters possessed a stress-related MBS-motif, CGTCA-motif, TGACG-motif regulatory element, and plant-immunity-related TC-rich element (Figure 9). In addition, specific cis-elements such as the LTR motif and TGA motif were found in the *CRK5* promoter region (Figure 9), suggesting *CRK5* is involved in low-temperature and phytohormone responses. Overall, the promoter sequences of *CRK* genes implied the involvement of *CRK* genes in various stress responses, for instance, ABA signal, low-temperature, and abiotic stimuli.

### 2.9. The Expression of CRK Genes Is in Response to Biotic and Abiotic Stresses

**Figure 10:**
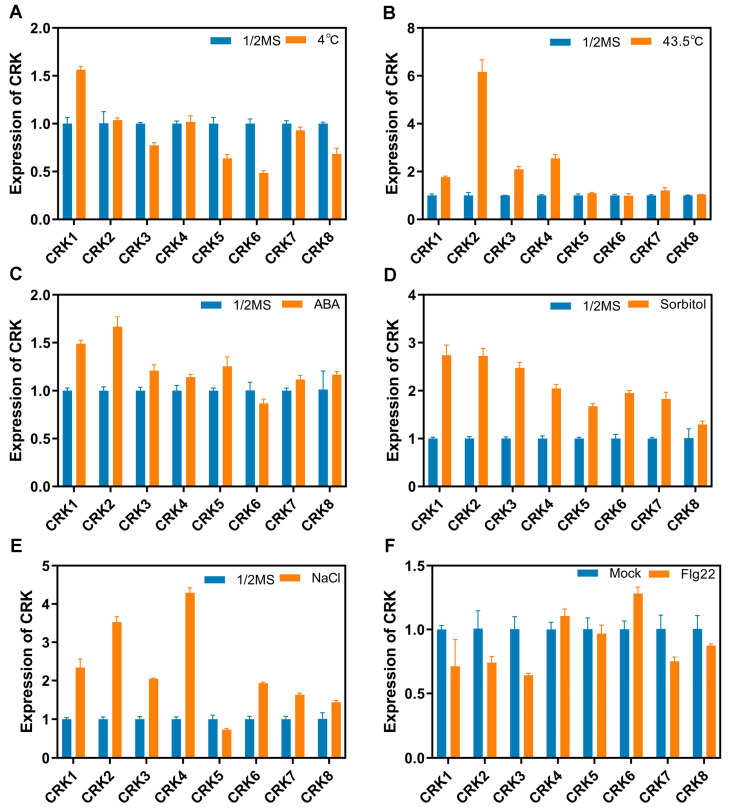
The expression of *CRKs* is in response to stress conditions. qPCR analysis was performed to assess expression of CRK genes under the following treatments: (**A**) 4 °C, (**B**) 43.5 °C, (**C**) 10 μM ABA, (**D**) 200 mM sorbitol, and (**E**) 200 mM NaCl for 24 h. (**F**) 1 μM flg22 in 1/2 MS liquid medium for 4 h. 1/2 MS liquid medium was used as control treatment.

The cis-element analysis of *CRK* promoters suggested that *CRKs* are engaged in various stress responses. We thus examined how *CRK* expression responded to different stress conditions, such as flg22, ABA, drought, salt, and high/low temperatures, by using qPCR. Low temperature highly induced *CRK1* and slight reduced *CRK5* and *6* (Figure 10A). *CRK2* was significantly prompted by high temperature (Figure 10B). ABA slightly enticed the expression of *CRK1* and *2* (Figure 10C). Almost all *CRK* genes were upregulated by sorbitol or NaCl treatment (Figure 10D,E). The presence of pathogenic elicitor, flag22, appeared not to obviously affect the expression of *CRKs* (Figure 10F). Consistent with cis-element analysis of *CRK* promoters, the qPCR results indicated *CRKs* are regulated by various stressed conditions at transcriptional levels.

### 2.10. Identification of CRK-Interacting Proteins by IP-MS

**Figure 11:**
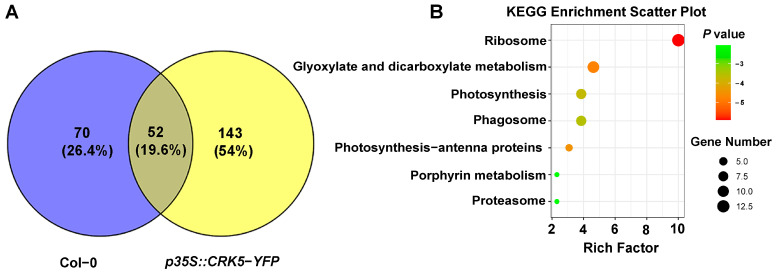

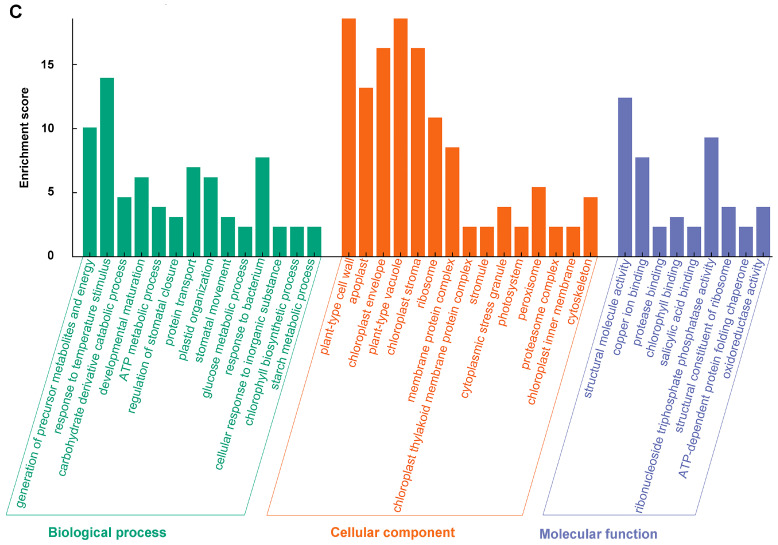
Mass spectrometry analysis of CRK5-interacting proteins. (**A**) Venn diagram showing the number of proteins enriched for *35S*::*YFP* in Col-0 vs. *35S*::*CRK5*-*YFP* in Col-0. (**B**) KEGG analysis of mass spectrometric data. (**C**) GO analysis and identification of mass spectrometric data. Predicting the most enriched molecular functions, biological processes, and cellular components. Y-axis: enrichment score, X-axis: enrichment factor.

To exert their functions, the kinases need to directly interact with their substrates for full phosphorylation. It, therefore, is important to identify the interacting components of the kinases to understand their functions. *CRK5* is so far the most characterized *CRK* gene, which has been shown to regulate several signaling events such as PIN2 regulation, embryogenesis, and an ROS-NO balance [30,31,32]. We thus used CRK5 to screen its interacting proteins. By using *CRK5*-*YFP* transgenic plants, we screened the interacting proteins of CRK5 through an IP-MS approach. In total, 143 proteins were identified (Figure 11A). Analysis using the Kyoto Encyclopedia of Genes and Genomes (KEGG) indicated that CRK5 is likely involved in metabolism, genetic information processing, and cell processes (Figure 11B). Gene ontology (GO) analysis suggested that the CRK5-interacting proteins are involved in biological processes, including low-temperature stress, plant immunity, and protein transportation (Figure 11C). CRK1, a homologous protein of CRK5, was identified in the CRK5-containing complex (Table S1), suggesting CRK kinases may form homo- or hetero-dimers to fulfill their functions. Moreover, an RLK, somatic embryogenesis receptor kinase 4 (SERK4), was found to be associated with CRK5 (Table S2). SERK4, together with its close homolog SERK4/BRI1-associated kinase 1 (BAK1), acts as a co-receptor for multiple ligand-binding receptors such as BRI1 and FLS2 to modulate plant growth and immunity [34]. Furthermore, several protein channels/transporters, such as plastid envelope ion channels 2 (PEC2), sugar transport protein 1 (STP1), ABC transporter of the mitochondrion 3 (ATM3), and karyopherin enabling the transport of the cytoplasmic hyl 1 (KETCH1), were found to interact with CRK5 (Table S2). The identification of RLK and channels/transporters as the components that bind to CRK5 dropped a hint that RLKs and transport proteins function upstream and downstream of CRK kinases, respectively.

## 3. Discussion

Despite numerous studies on *CPK* in regulating growth, development, and stress responses in plants, the functions of *CRK*, the closest homolog of *CPK*, are still largely unknown. In this study, we investigated *CRK* genes in plant genomes and found that *CRK* only exists in land plants, indicating *CRK* is likely divided from *CPK* at the early stages of plant evolution. In Arabidopsis, eight *CRK* genes were identified. We provided evidence that *CRKs* are broadly expressed during plant growth and stress adaptions. We also showed that Arabidopsis CRK possesses kinase activity and, importantly, is Ca^2+^-independent. By utilizing a proteomic approach, we identified a number of candidate proteins that potentially interact with CRK5. Among the CRK5-interacting proteins, RLK and transporters were pinpointed. It is thus conjectured that RLKs and channels/transporters function upstream and downstream of CRKs, respectively.

*CRK* is only found in land plants starting from moss but not in algae. In contrast, *CPK* is found in land plants and algae. This result demonstrated that *CRK* was originally derived from *CPK*, which likely occurred during the evolutionary stages when ancient plants relocated from the oceans to the lands. Future studies will focus on the potential selection pressures that drove *CRK*–*CPK* separation and caused the origin of *CRK*. Our results indicated that CRK is spontaneously active regarding its autophosphorylation ability. The presence of Ca^2+^ was unable to affect the kinase activity of CRK. Even though this can be explicated by the degenerative EF-hands in CRK, it remains unknown whether the CaM-like domain of CRK still functions as a regulatory domain. Although the generative EF-hands are not capable of directly binding to Ca^2+^, it cannot be excluded that they can recruit additional components to form complexes or associate with the substrate proteins to determine CRK specificities. Considering that the CaM-like domains in a number of proteins were found to interact with CaM or CaM domain [35,36], the CaM-like domain of CRK may act as the target of Ca^2+^ decoding components such as CaM and CBL, which allows indirect involvement of CRK in Ca^2+^-mediated signaling.

The kinase domain of CPK appears to be repressed by the AI-JD via direct association under Ca^2+^-free conditions. Despite the lack of Ca^2+^ binding affinity, CRK still contains an AI-JD with unknown functions. CRK appears to be spontaneously active, in which Ca^2+^ is not involved. It thus will be interesting to explore whether the AI-JD of CRK still interacts with its kinase domain if AI-JD inhibits the kinase domain of CRK and whether the phosphorylation of AI-JD results in their disassociation. If the AI-JD of CRK fails to interact with the kinase domain, the comparison analysis of the AI-JDs between CPK and CRK will provide additional insights into the molecular mechanisms underlying the auto-inhibition and activation of CPK and CRK.

CPK is activated and regulated by Ca^2+^ oscillations. Moreover, CPK is activated by other kinases, such as RLKs and other CPKs, through phosphorylation [37,38,39,40,41]. Given that Ca^2+^ signals activate CPK but not CRK, it is important to investigate the upstream components that activate CRK in future studies. Of note, RLK SERK4 was found to interact with CRK5. SERK4 and SERK3/BAK1 were previously identified as essential co-receptors for multiple ligand-binding receptors-mediated pathways such as brassinosteroid (BR), pattern-triggered immunity (PTI), and effector-triggered immunity (ETI) [42,43,44,45,46,47]. The identification of RLK as the interacting component of CRK led to an assumption that RLK perceives extracellular ligands such as phytohormones and pathogen-associated molecular patterns (PAMPs) and subsequently activates CRKs to initiate intracellular signaling.

CPK activity is modulated by Ca^2+^ signatures, which often reflect instantaneous exogenous and endogenous events [48]. Thus, the substrates usually include transporters and channels that mediate rapid ion fluxes in response to acute intercellular and intracellular stimuli. The transphosphorylation of channels and transporters mediated by CPK causes conformational change, leading to activation/inactivation of their transport abilities. It has been well established that CPK phosphorylates a variety of channels and transporters that mediate the transport of cations, anions, H_2_O, and phytochromes [49,50]. Intriguingly, various transporters were identified to be associated with CRK5 during proteomic assay. It suggested CRK, similar to CPK, phosphorylates transporters and channels to manipulate ion/membrane potential/pH homeostasis. In this study, RLK and channels/transporters were identified to be associated with CRK5. Given that Ca^2+^ is not capable of directly activating CRK, it will be interesting to explore whether RLK can function upstream of CRK. We speculated that distinct from CPK, which is activated by Ca^2+^ signals, CRK can be activated by PM-localized RLK upon apoplastic ligand perceptions to regulate the activities of channels and transporters, leading to rapid cellular responses.

Most *CPKs* were found to play their roles under stressed conditions. Hence, very few *cpk* mutant plants display obvious developmental defects under normal growth conditions. Most *cpk* mutants exhibit phenotypes different from WT plants only when they are subjected to stress treatments. This reminds us that genetic analysis on *CRK* may require examining the *crk* mutant plants by using different abiotic and biotic conditions. In addition, due to gene redundancy, high-order mutant plants for *CRK* need to be generated and examined for genetic analysis. The expression patterns of *CRK* presented in this study may provide information for constructing *crk* multiple mutants in future studies. Furthermore, the functional analyses of *CRK* genes may rely on multiple strategies, including proteomic and genomic methods. For *crk* mutants, RNAseq can be utilized in different treatments, such as phytohormones and stressed conditions, to identify potential CRK-regulated genes, which may indicate the possible signaling pathways in which the CRKs are involved. In addition, proteomic approaches will help to screen CRK-interacting components.

Compared to CPK, which is extensively involved in all aspects of plant growth and stress adaptions, CRK is barely functionally analyzed. In this study, we systematically characterized Arabidopsis *CRK* genes in terms of genetic and biochemical futures, which provides an additional understanding of Ca^2+^-related and Ca^2+^-unrelated signaling pathways mediated by protein kinases in plants.

## 4. Materials and Methods

### 4.1. Plant Materials and Growth Conditions

The Col-0 accession of *Arabidopsis thaliana* was used as the wild type (WT) in all experiments conducted in this study. The plants were transformed using the *Agrobacterium tumefaciens*-mediated floral dip method [51]. Arabidopsis plants were cultivated in soil within a greenhouse under long-day conditions (16 h light/8 h dark, 22 °C). *Nicotiana benthamiana* was grown in soil in a plant incubator at 28 °C under a 16 h light/8 h dark photoperiod.

### 4.2. Molecular Cloning and Construction of Transgenic Plants

The sequences of primers used for vector construction in this study are listed in Supplemental Table S3. The PCR products were recombined into the *pDONR* vector via the Gateway BP reaction, followed by the Gateway LR reaction to construct the destination vectors (Invitrogen, Waltham, MA, USA). To generate of overexpression plants, the coding sequences (CDS) of CRK were recombined into the binary vector *pBIB-BASTA-35S-GWR-YFP*. For GUS staining, the promoter sequences of approximately 2.0 kb of *CRK* genes were cloned into the binary vector *pBIB*-*BASTA*-*GWR*-*GUS*. The destination vectors were then transformed into plants using the floral dip method with *A. tumefaciens* strain GV3101 [51].

### 4.3. Database Search and Sequence Retrieval

The protein sequences of AtCRK and AtCPK were downloaded from the Arabidopsis information resource (TAIR) (https://www.arabidopsis.org/) URL (accessed on 23 March 2025). Since the protein sequences of CRK and CPK are highly conserved in the N-segment and kinase domains, thus affecting the homologous protein search, we only used the more divergent C-terminal region for comparison. The protein sequences of CRK from other species were retrieved from Phytozome (https://phytozome-next.jgi.doe.gov/) URL (accessed on 23 March 2025) and National Center for Biotechnology Information (NCBI) (https://www.ncbi.nlm.nih.gov/) (E ≤ 10^−5^) URL (accessed on 23 March 2025). The CDS and promoter sequences of *CRK* were obtained from the TAIR (https://www.arabidopsis.org/) URL (accessed on 23 March 2025).

### 4.4. Phylogenetic Analysis and Chromosomal Distributions of CRK Genes

The phylogenetic tree was constructed to elucidate the evolutionary relationship of CRK proteins among different species. The protein sequences were aligned using ClustalW to generate a Clustal file [52]. The generated Clustal files were then converted to the MEGA format using MEGA11.0.10 software [53]. Unrooted phylogenetic trees were generated using the maximum likelihood method in MEGA11.0.10, with bootstrap tests conducted using 1000 replicates. The trees were visualized using the iTOL website (https://itol.embl.de/) URL (accessed on 23 March 2025). The chromosomal distribution of *CRK* genes in the Arabidopsis genome was analyzed by using the online tool MG2C (http://mg2c.iask.in/mg2c_v2.1/) URL (accessed on 23 March 2025) [54].

### 4.5. Multiple Sequence Alignment and Myristoylation Site Prediction

Multiple sequence alignments of CRK and CPK proteins were performed separately to identify conserved domains and motifs. GENEDOC 2.7.0 software was used to perform the multiple sequence alignment. The conserved domains and motifs were predicted using Motif Scan (https://myhits.sib.swiss/cgi-bin/motif_scan) URL (accessed on 28 March 2025). The myristoylation sites of CRK and CPK proteins were predicted using the NMT Predictor (https://mendel.imp.ac.at/myristate/SUPLpredictor.htm) URL (accessed on 28 March 2025).

### 4.6. Model Performance Analysis and Visualization

The prediction of CRK and CPK protein structures was performed using AlphaFold3 (https://golgi.sandbox.google.com/) URL (accessed on 28 March 2025) [55]. In addition, the differences in the binding sites of CRK and CPK to metal ions were analyzed. The structure visualizations were made in PyMOL 2.5.x software

### 4.7. Promoter Sequence Analysis and Physicochemical Properties of CRK Proteins

The promoter sequences of the *CRK* genes were downloaded from the TAIR (https://www.arabidopsis.org/) URL (accessed on 28 March 2025). The promoter sequences are approximately 2.0 kb. PlantCARE (https://bioinformatics.psb.ugent.be/webtools/plantcare/html/) URL (accessed on 28 March 2025). was used to analyze the promoter elements. The results are visualized using TBtools 2.0 software. In addition, the physicochemical properties of CRK proteins were analyzed using TBtools 2.0 software [56].

### 4.8. Subcellular Localization Assay

Subcellular localization of CRKs was determined by observing the YFP fluorescence signal. The CDS of *CRKs* were cloned into the *pBIB-BASTA*-*35S*-*YFP* vector with the cauliflower *mosaic virus 35S (CaMV35S)* promoter. The *35S*::*YFP* control construct and *35S*::*CRK*-*YFP* constructs were transiently expressed in *N. benthamiana.* Tobacco protoplasts were prepared as previously described [57]. The plasma membrane of chloroplasts was labeled with FM4-64, and YFP signals were detected using a confocal microscope (Leica/Stellaris 5) with laser excitation at a wavelength of 514 nm and between 520 and 550 nm and an excitation laser at a wavelength of 561 nm and between 570 and 630 nm to detect FM4-64 signals [57].

### 4.9. Expression and Purification of Recombinant CRK in Escherichia coli

Recombinant *MBP*-*CRKs* were expressed in *E. coli* (*Rosetta*). Protein induction and purification were performed as previously described [58]. The glutathione agarose beads (Sangon Biotech, Shanghai, China) were used according to the manufacturer’s manual.

### 4.10. In Vitro Kinase Assays

For phosphorylation assays using protein blotting, full-lengths of *CRK*s were PCR amplified and cloned into the *pDEST15* vector with an MBP tag (Invitrogen, 11802014) to generate fusion proteins of MBP-CRK3, MBP-CRK5, and MBP-CRK8. Proteins expressed in *E. coli* were purified using glutathione agarose beads (Sangon Biotech, C600031) according to the manufacturer’s instructions. For the phosphorylation assay, reaction conditions reference [54]. For Pro-Q assays, for sample preparation and reaction conditions, please refer to Sun [59]. Imaging with a PharosFX molecular imager (Bio-Rad, Hercules, CA, USA) instrument.

### 4.11. GUS Staining

Different developmental stages and different tissues of transgenic plants were collected and stained. The tissues were first incubated in rinsing solution (34.2 mM Na_2_HPO_4_, 15.8 mM NaH_2_PO_4_, 0.5 mM K_3_Fe(CN)_6_, and 0.5 mM K_4_Fe(CN)_6_ 3H_2_O) for 5 min, then incubated in staining solution (34.2 mM Na_2_HPO_4_, 15.8 mM NaH_2_PO_4_, 0.5 mM K_3_Fe(CN)_6_, 0.5 mM K_4_Fe(CN)_6_ 3H_2_O, and 2 mM X-Gluc) at 37 °C for appropriate time. After staining, the plant tissues were immersed in 30%, 50%, 75%, and 95% ethanol for 1 h in succession and then immersed in 75% ethanol. After destaining, the tissues were observed under a stereomicroscope (Leica M165C).

### 4.12. RT-qPCR

To quantify *CRK* gene expression at different time points, total RNA extraction in Col-0 was performed with RNAprep Pure Plant Kit (TIANGEN, Beijing, China) according to the manufacturer’s instructions. Two micrograms of total RNA were used for reverse transcription using the Reverse Transcription System (Takara, Shiga, Japan). The PCR program was run on the Step One Plus Real Time PCR system (Applied Biosystems, Waltham, MA, USA). *ACTIN2* (*ACT2*) expression was used as an internal control to normalize all data. Each experiment was performed with at least three independent biological replicates.

### 4.13. Mass Spectrometry Analysis

For the IP experiment, the transgenic seedlings were cultured in a liquid MS medium with gentle shaking for 10 d. The seedlings were ground in liquid nitrogen and lysed in extraction buffer, which includes 10 mM HEPES (pH 7.5), 100 mM NaCl, 1 mM EDTA, 10% glycerol, 1% TritonX-100, 20 mM NaF, 1 mM PMSF, 1 × protease inhibitor. The transparent supernatant was obtained by multiple centrifugation (6000× *g*, 4 °C, 10 min). The supernatant was incubated with anti-GFP beads (KTSM1301, Alpalife, Shenzhen, China) for 3 h at 4 °C with gentle shaking. The beads were washed five times using extraction buffer containing 0.2% TritonX-100 and then boiled with 2 × SDS loading buffer (325 mM Tris, pH 6.8, 10% (*w*/*v*) SDS, 50% (*v*/*v*) glycerol, 25% (*v*/*v*) β-mercaptoethanol, and 0.05% (*w*/*v*) bromophenol blue) for 5 min. The proteins were separated in 8% SDS-PAGE gel. The antibodies in this study were α-GFP (11814460001, Roche, Mannheim, Germany) antibodies. For the mass spectrometry experiment, the sample preparation is referred to as Sun [59]. The samples were analyzed using an Orbitrap Fusion Lumos mass spectrometer (Thermo Fisher Scientific, Waltham, MA, USA) connected to an EASY-nLC 1200 system. Proteome Discoverer Daemon 2.2 software (Thermo Fisher Scientific) was used for data analysis.

### 4.14. Data Acquisition and Statistics

To analyze the functional characteristics of CRK protein, GO functional annotation and protein enrichment analysis were performed using Metascape (https://metascape.org/gp/index.html#/main/step1) URL (accessed on 28 March 2025). For KEGG pathway analysis, KEGG (https://www.genome.jp/kegg/pathway.html) URL (accessed on 28 March 2025) was searched. Visualizations of the results were generated online (https://www.bioinformatics.com.cn/) URL (accessed on 28 March 2025).

## Figures and Tables

**Table 1:** Physicochemical properties of CRK proteins.

Protein Name	Number of Amino Acid	Molecular Weight	Theoretical pI	Instability Index	Aliphatic Index	Grand Average of Hydropathicity
CRK1	576	64,315.06	8.76	47.87	84.97	−0.232
CRK2	599	67,207.07	9.06	53.91	86.49	−0.340
CRK3	595	66,600.05	8.96	47.04	82.64	−0.395
CRK4	594	66,513.96	8.46	49.52	79.33	−0.369
CRK5	632	70,478.64	9.01	47.31	81.99	−0.312
CRK6	594	66,371.73	8.33	52.60	81.99	−0.328
CRK7	577	64,547.31	8.84	51.55	88.54	−0.245
CRK8	606	67,972.80	9.15	51.59	81.65	−0.352

**Table 2:** Statistics of conserved motifs of EF-hands of CPK and CRK in Arabidopsis.

EF-Hand	CPK	CRK
1st	D-x-D, D-x-N, D	None
2nd	D-x-D, D-x-N, D	None
3rd	D-x-D, D-x-N	None
4th	D-x-D, D-x-N	None

## Data Availability

The data that support the findings of this study are available from the corresponding author upon reasonable request.

## Supplementary Materials

The following supporting information can be downloaded at https://www.mdpi.com/article/10.3390/ijms26073297/s1.
