# Supplementary material for: Genome-Wide Investigation of CPK-Related Kinase (CRK) Gene Family in Arabidopsis thaliana
Source: Int J Mol Sci. 2025 Apr 2;26(7):3297. doi: 10.3390/ijms26073297 (PMC11989534; doi:10.3390/ijms26073297)
Supplement: Supplementary file 1 [file ijms-26-03297-s001.zip › Supplementary.pdf]

Appendix

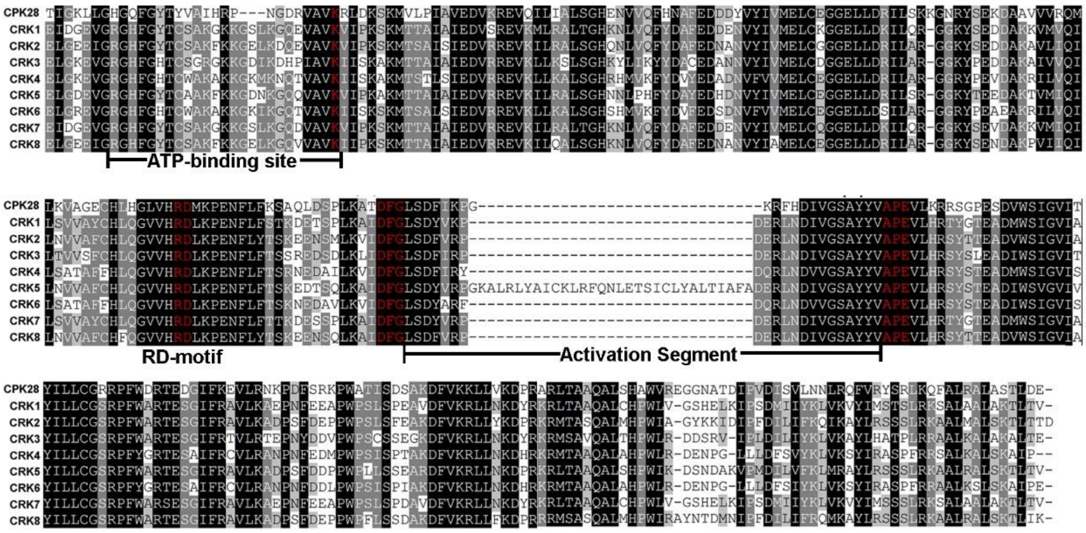

Supplemental 1. Structure based sequence alignment of the activation segment of active protein kinase structures. The kinase domains of CRK and CPK include ATP-binding site, with core lysine/K indicated in red, RD motif, and activation segment (between DFG tripeptide motif and APE tripeptide motif).

A

Taxonomy	Name	CPK	CRK
Chlorophyta	Chlamydomonas reinhardtii	+	-
	Chromochloris zofingiensis	+	-
Rhodophyta	Chondrus crispus	+	-
	Galdieria sulphuraria	+	-
Bryophyta	Marchantia polymorpha	+	+
	Physcomitrium patens	+	+

B

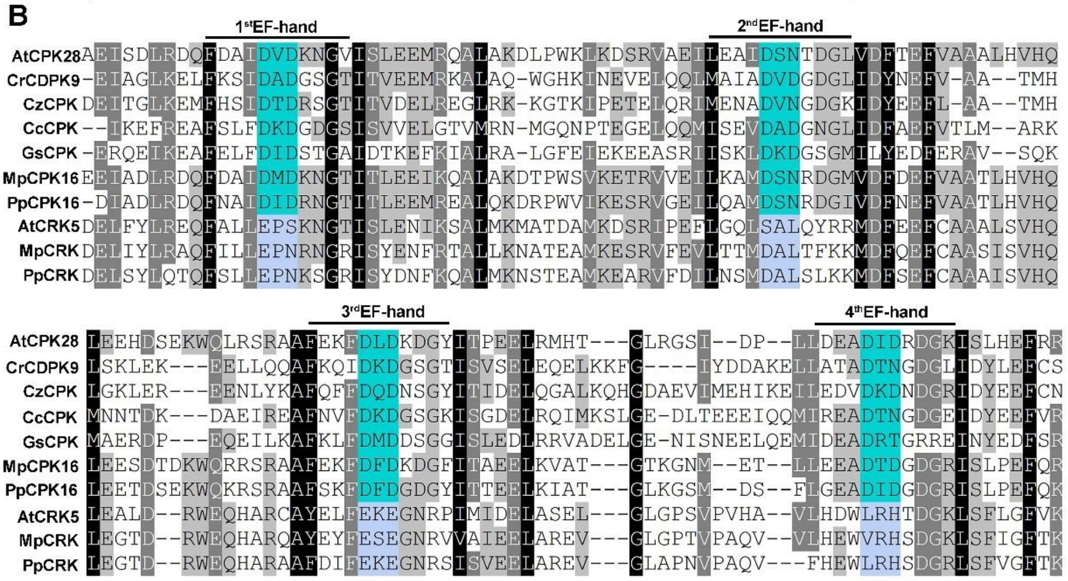

Supplemental 2. Alignment of and the orthologous proteins of Arabidopsis CRK/CPK in Chlorophyta, Rhodophyta, and Bryophyta. (**A**) Statistics of individual CRK and CPK for different species. + indicates presence; - indicates absence. (**B**) The presence of conserved EF-hand regions of CPK in different species. CPK is present in Chlorophyta, Rhodophyta and Bryophyta, while CRK is only present in Bryophyta. Medium turquoise indicates a conserved D-X-D motif, and light steel blue indicates degenerative EF-hand.

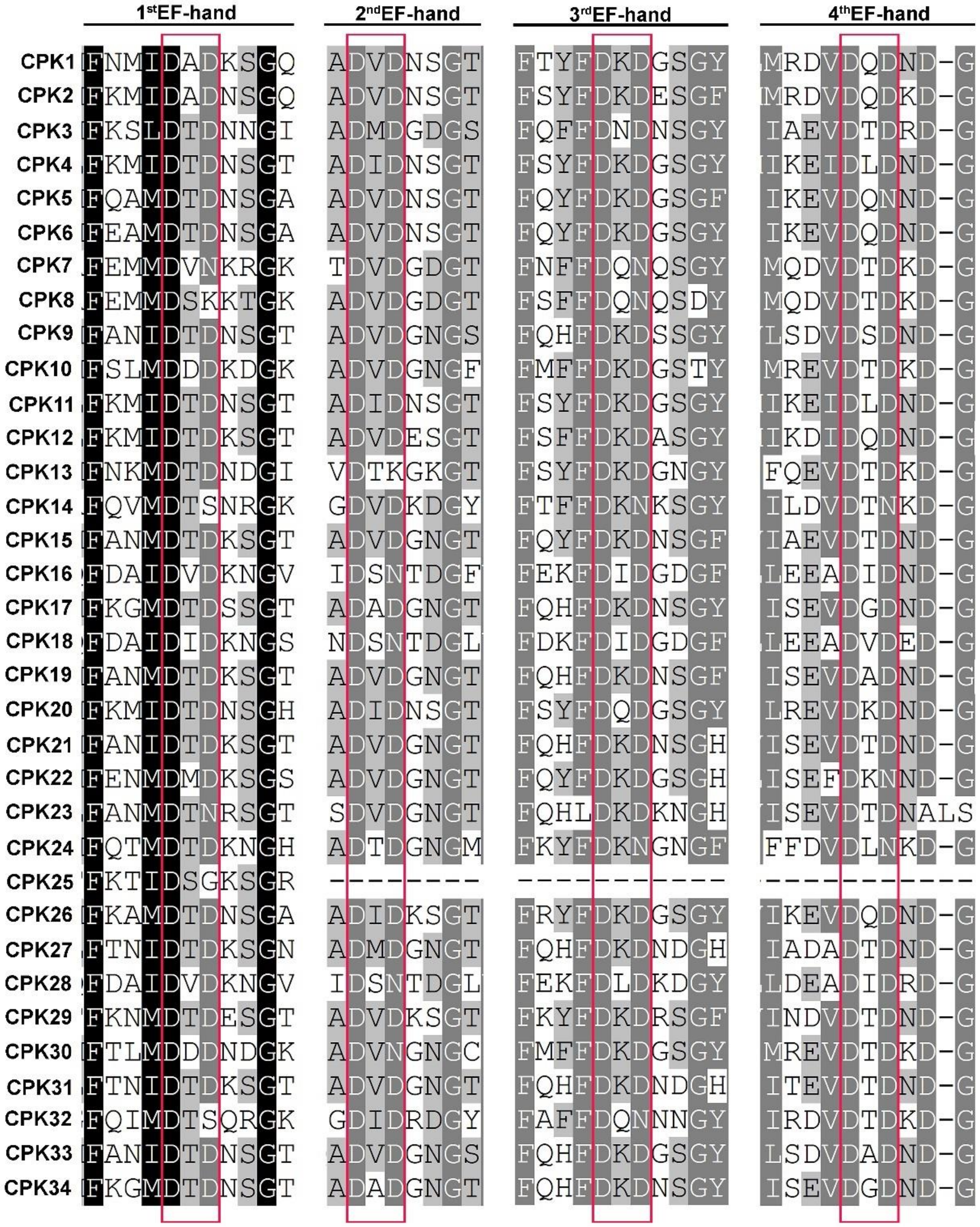

Supplemental 3. The presence of conserved EF-hands in all Arabidopsis CPKs. Multiple sequence alignment of C-terminal sequences of CPK proteins. CPK proteins contain EF-hand domains with conserved D-x-D or D-x-N motifs.

Supplemental Table 1. KEGG Pathway Enrichment

KEGG Pathway															
ath00710		ath03010		ath00196		ath00630		ath00195		ath04145		ath00860		ath03050	
Carbon fixation in photosynthetic organisms		Ribosome		Photosynthesis		Glyoxylate and dicarboxylate metabolism		Photosynthesis		Phagosome		Porphyrin metabolism		Proteasome	
**Gene ID**	**Name of**	**Gene ID**	**Name**	**Gene ID**	**Name**	**Gene ID**	**Name**	**Gene ID**	**Name**	**Gene ID**	**Name**	**Gene ID**	**Name**	**Gene ID**	**Nar**
AT2G41140	CRK1	AT2G39460	RPL23A	AT1G15820	LHCB6	AT1G62380	ACO2	AT4G04640	ATPC1	AT3G18780	ACT2	AT1G74470	CHLP	AT5G20000	RPT
AT1G79550	PGK	AT1G26880	RPL34A	AT2G05100	LHCB2.1	AT5G56720	MDH3	AT3G58730	VHA-D	AT3G58730	VHA	AT3G56940	CRD1	AT3G22110	PA
AT5G56720	MDH3	AT2G37270	RPS5B	AT5G01530	LHCB4.1	AT3G47520	MDH	ATCG00020	PSBA	AT2G34250		AT4G27440	PORB	AT3G05530	RPT
At3G47520	MDH	AT4G18100	RPL32A	AT1G61520	LHCA3	AT3G06650	ACLB-1	AT2G33040	ATP3	AT1G52280	RABG3d				
AT3G17390	MTO3	AT3G60770	RPS13A			AT3G17390	MTO3	AT4G12800	PSAL	AT3G12110	ACT11				
AT4G39980	DHS1	ATMG00290	RPS4			AT4G18360	GOX3	AT2G24520	HA5						
AT4G18360	GOX3	AT3G11250	UL10X			AT4G01850	SAM-2	AT2G41700	ATPB						
AT1G12900	GAPA-2	AT1G04480	UL14X			AT5G14780	FDH	ATCG00280	PSBC						
AT4G01850	SAM-2	AT5G56710	EL31X			AT4G35090	CAT2								
AT5G14780	FDH	AT3G07040	RPS3			AT5G37600	GSR 1								
AT3G52930	FBA8	AT5G27770	EL22Y			AT5G11520	ASP3								
AT4G35090	CAT2	AT3G52580	US11X												
AT5G37600	GSR1	AT1G12220	RPS5												
AT2G36530	LOS2														
AT4G15530	PPDK														
AT5G11520	ASP3														

Supplemental Table 2. Go Biological Processes Enrichment

GO Biological Processes													
GO:0006091	GO:0009266	GO:1901136	GO:0021700	GO:0046034	GO:0090333	GO:0015031	GO:0009657	GO:0010118	GO:0006006	GO:0009617	GO:0071241	GO:0015995	GO:0005
generation of precursor metabolites and energy	response to temperature stimulus	carbohydrate derivative catabolic process	developmental maturation	ATP metabolic process	regulation of stomatal closure	protein transport	plastid organization	stomatal movement	glucose metabolic process	response to bacterium	cellular response to inorganic substance	chlorophyll biosynthetic process	starch metabo proces
**Gene ID**	**Gene ID**	**Gene ID**	**Gene ID**	**Gene ID**	**Gene ID**	**Gene ID**	**Gene ID**	**Gene ID**	**Gene ID**	**Gene ID**	**Gene ID**	**Gene ID**	**Gene I**
AT3G26650	AT3G09440	AT1G79550	AT3G18780	AT1G79550	AT5G23060	AT2G04030	AT3G47520	AT1G47128	AT3G26650	AT1G79550	AT5G23060	AT1G74470	AT5G49
AT1G79550	AT3G18780	AT3G09790	AT5G60660	AT1G26570	AT1G47128	AT3G11130	AT5G46290	AT2G04030	AT1G12900	AT1G47128	AT1G62380	AT3G56940	AT5G24
AT1G15820	AT2G41140	AT1G26570	AT3G14310	AT4G04640	AT2G05100	AT3G47520	AT5G58270	AT3G11130	AT3G52930	AT3G14310	AT4G20260	AT4G27440	AT5G48
AT4G04640	AT3G26650	AT4G04640	AT5G49190	AT3G47520	AT3G16640	AT1G11260	AT3G56940	AT2G33380		AT3G47520			
AT4G01050	AT5G23060	AT3G09260	AT3G46520	AT2G33040	AT5G25980	AT2G21390	AT2G33150	AT5G56030		AT2G13790			
AT1G71500	AT1G79550	AT2G33040	AT1G03880	AT3G06650		AT3G08530	AT4G01800	AT2G05100		AT4G39980			
AT3G06650	AT2G04030	AT3G06650	AT5G44120	AT3G17390		AT5G19820	AT1G06950	AT4G20260		AT4G18360			
AT4G35250	AT1G15820	AT5G24300	AT3G16640	AT4G39980		AT4G01800	AT2G33800	AT3G16640		AT2G33380			
AT1G12900	AT3G09260	AT3G22110		AT2G33150		AT1G06950				AT3G54560			
AT2G05100	AT2G39460	AT3G16410		AT2G33380		AT3G08580				AT5G56030			
AT5G01530	AT3G47520	AT3G05530		AT3G52930		AT5G20010				AT3G11250			
AT3G52930	AT3G17390	AT3G52930		AT5G37600						AT5G20010			
AT2G36530	AT2G33380	AT2G36530		AT2G36530						AT4G20260			
AT1G61520	AT3G54560	AT5G25980		AT4G21210						AT5G25980			
AT5G48300	AT4G01800			AT5G11520						AT4G20830			
	AT5G56030												
	AT2G05100												
	AT4G35090												
	AT4G20260												
	AT2G36530												
	AT1G61520												
	AT5G48300												
	AT2G33800												
