# Supplementary figures and images for: Genome-Wide Investigation of CPK-Related Kinase (CRK) Gene Family in Arabidopsis thaliana
Source: Int J Mol Sci. 2025 Apr 2;26(7):3297. doi: 10.3390/ijms26073297 (PMC11989534; doi:10.3390/ijms26073297)

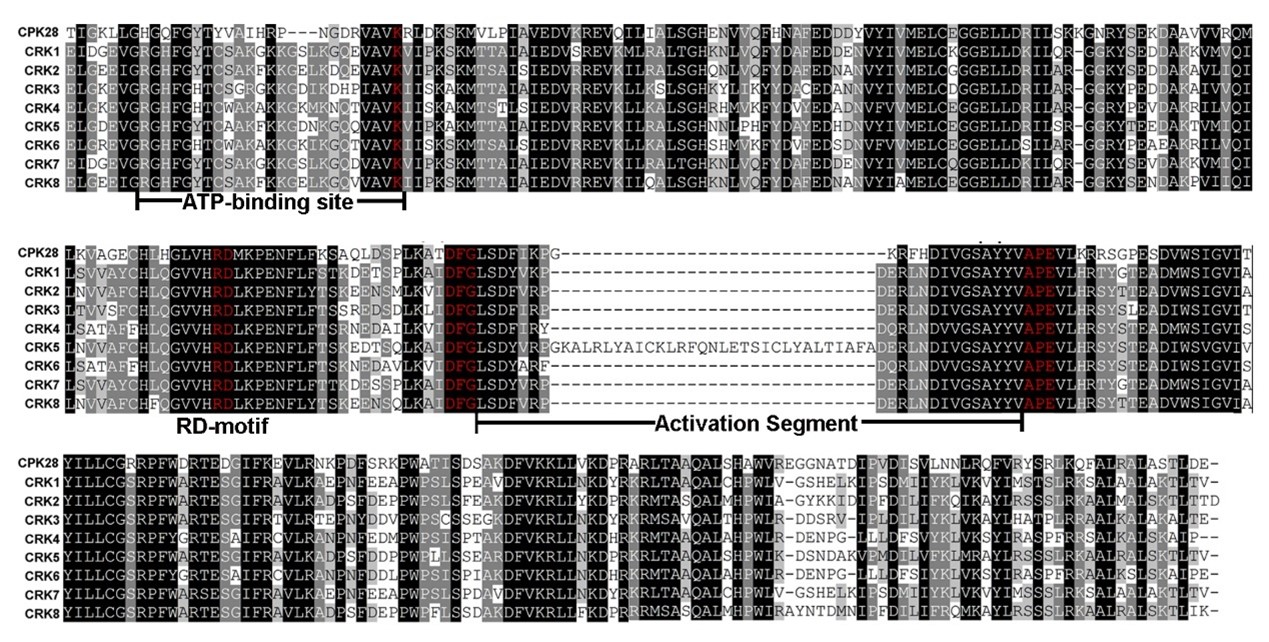

Supplement: Supplementary file 1 [file ijms-26-03297-s001.zip › Figure S1.jpg]

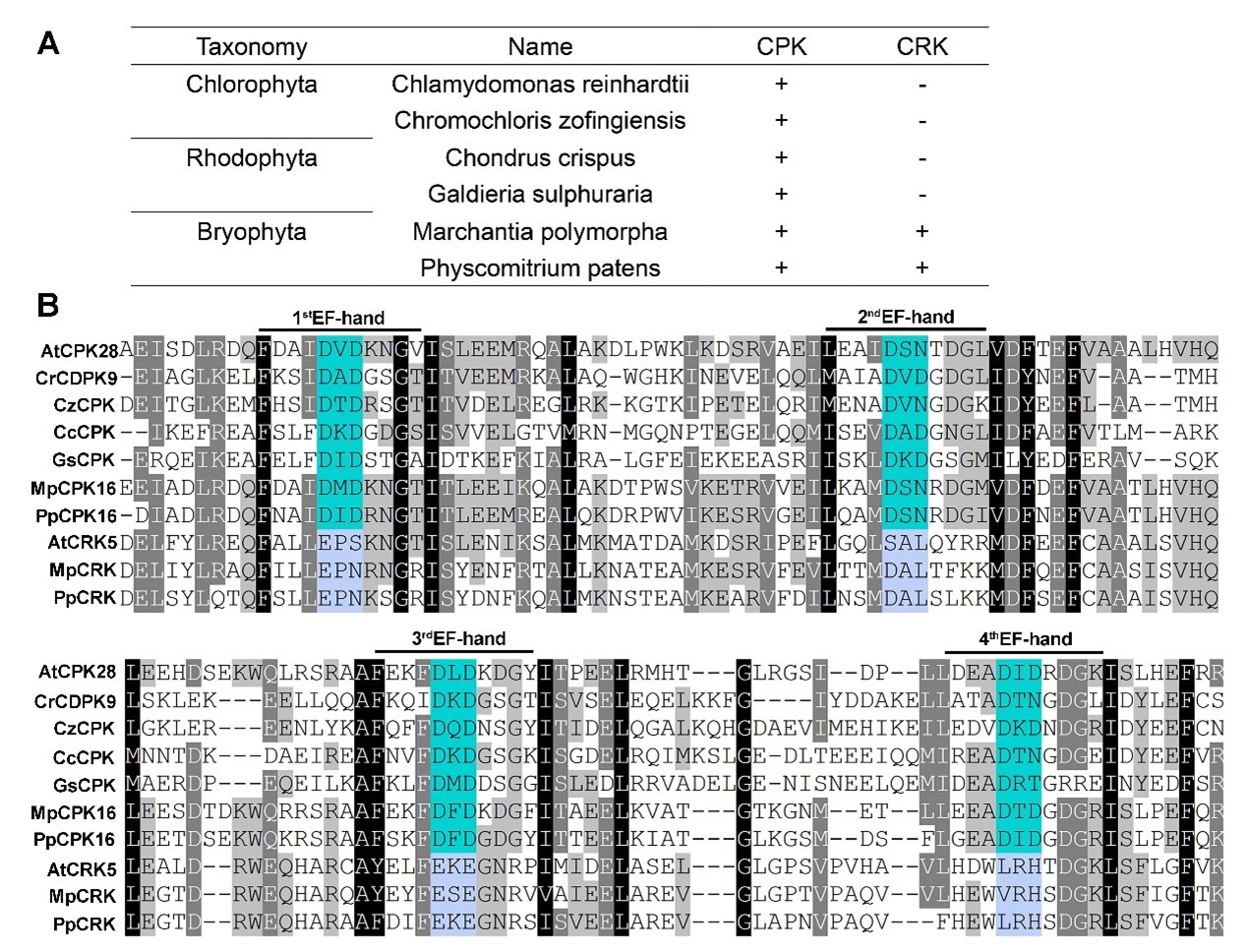

Supplement: Supplementary file 1 [file ijms-26-03297-s001.zip › Figure S2.jpg]

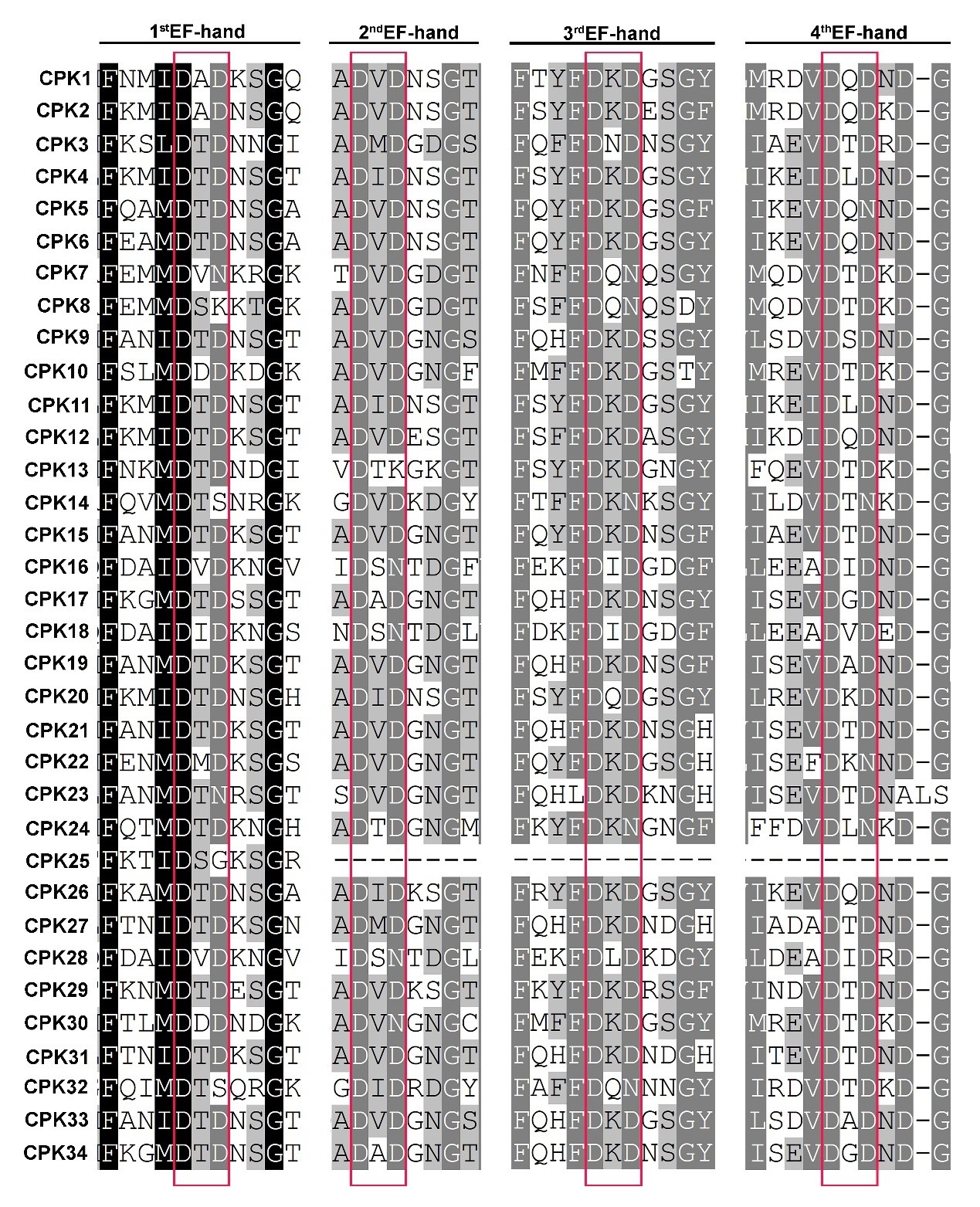

Supplement: Supplementary file 1 [file ijms-26-03297-s001.zip › Figure S3.jpg]

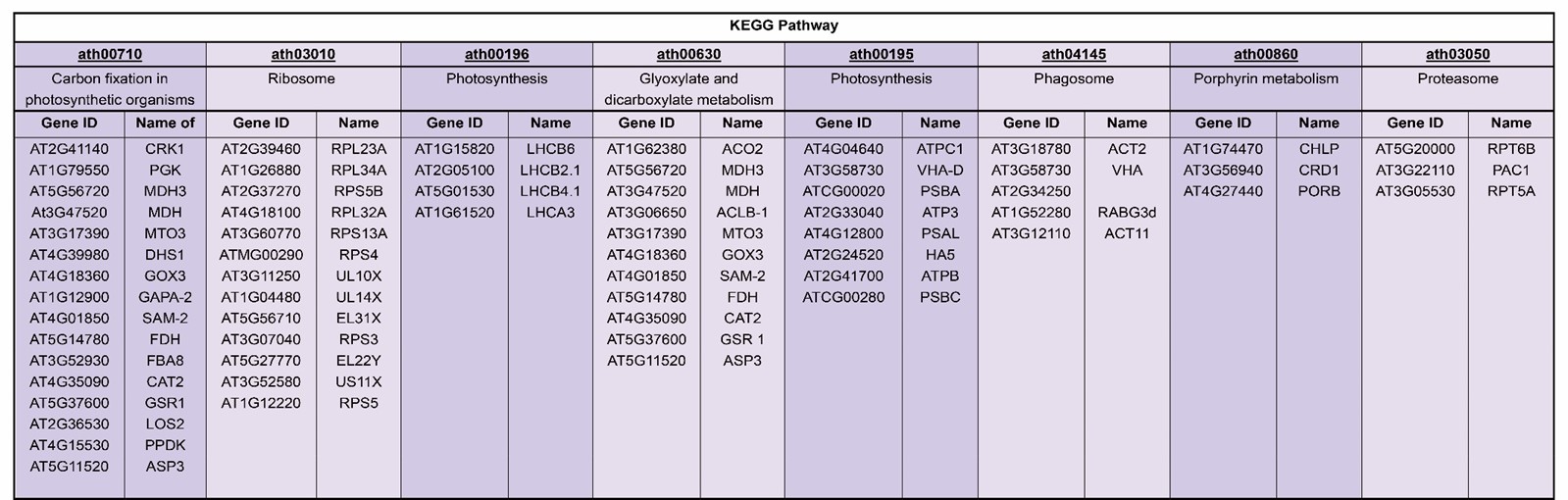

Supplement: Supplementary file 1 [file ijms-26-03297-s001.zip › Table S1.jpg]

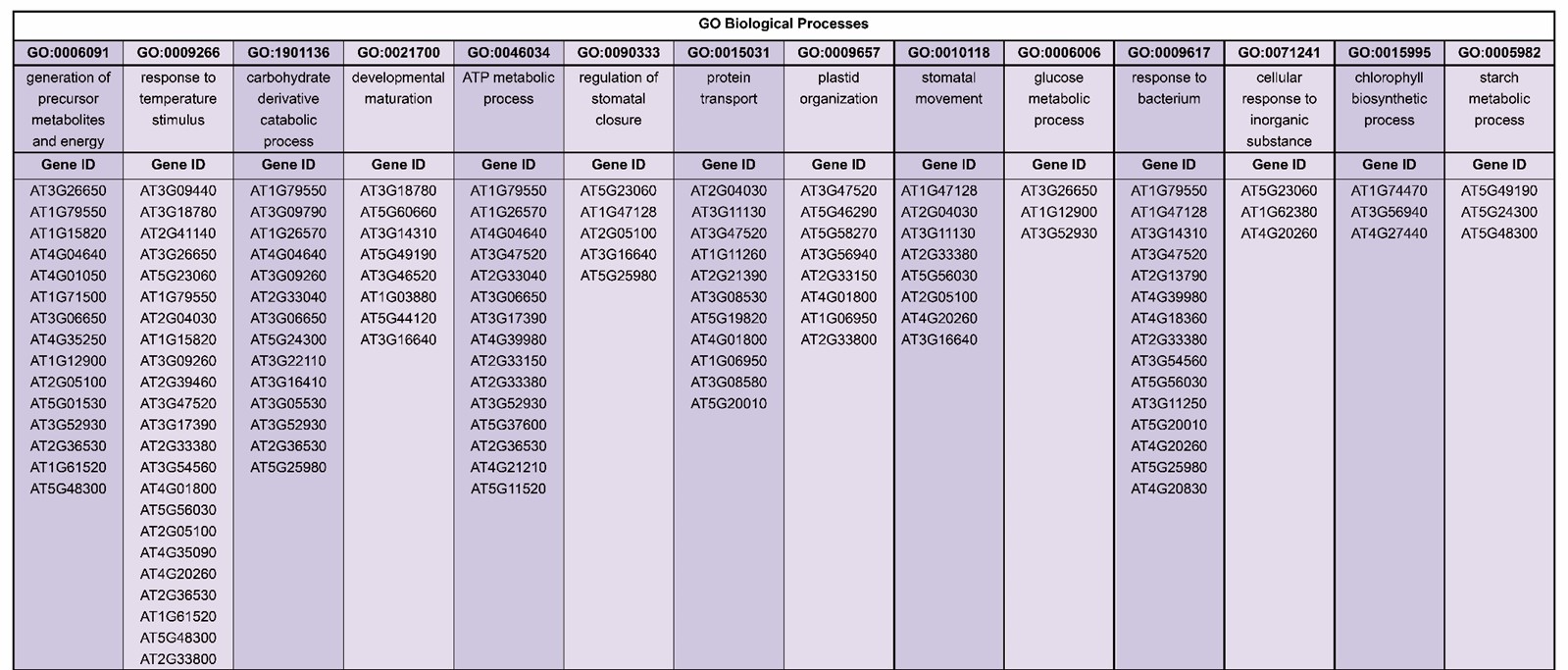

Supplement: Supplementary file 1 [file ijms-26-03297-s001.zip › Table S2.jpg]
